# Early variations in lymphocytes and T lymphocyte subsets are associated with radiation pneumonitis in lung cancer patients and experimental mice received thoracic irradiation

**DOI:** 10.1002/cam4.2987

**Published:** 2020-03-24

**Authors:** Pu Zhou, Lu Chen, Dong Yan, Changlin Huang, Guangpeng Chen, Zhiyi Wang, Liangzhi Zhong, Wen Luo, Diangang Chen, Chui Chun, Shushu Zhang, Guanghui Li

**Affiliations:** ^1^ Institute for Cancer Research in People's Liberation Army Xinqiao Hospital Army Medical University Chongqing China; ^2^ Institute for Pathology Xinqiao Hospital Army Medical University Chongqing China; ^3^ Institute for Radiology Xinqiao Hospital Army Medical University Chongqing China

**Keywords:** lung cancer, lymphocytes, radiation pneumonitis, T lymphocyte subsets

## Abstract

There were no ideal markers to predict the development of radiation pneumonitis (RP). We want to investigate the value of variations of lymphocytes and T lymphocyte subsets in predicting RP after radiotherapy (RT) of lung cancer based on previous clinical findings. A total of 182 lung cancer patients who received RT were retrospectively analyzed. Circulating lymphocytes and T lymphocyte subsets were measured before, during, and after RT. Patients were evaluated from the start of RT to 6 months post‐RT. A mice model with acute radiation‐induced lung injury was established and circulating lymphocytes were measured weekly until 8 weeks after irradiation. Univariate and multivariate analyses were adopted to identify risk factors of RP. Lymphocyte levels significantly decreased (*P* < .001) in patients before RP symptoms developed that also was able to be seen in the mice model and the values recovered during remission of symptoms. The decrease in lymphocyte count reflected the severity of RP. Meanwhile, CD4^+^ T lymphocyte count was significantly lower during the occurrence of symptoms in patients with RP than in those without RP (*P* < .001), and it improved along with RP recovery. Levels of lymphocytes and CD4^+^ T lymphocyte subsets proved as independent predictors of RP. Here we showed that lower peripheral blood levels of lymphocytes and CD4^+^ T lymphocyte were associated with an increased risk of RP, which was validated by this mice model, and thus are associated with differences in radiation‐induced lung toxicity among individuals and help identify those who are susceptible to developing RP after RT.

## Graphical abstract

We found lower levels of lymphocyte and CD4^+^ T lymphocyte linked with radiation pneumonitis and dynamic change of lymphocyte and CD4^+^ T lymphocyte predict radiation pneumonitis after 182 cases of lung cancer patients with radiation pneumonitis were retrospectively analyzed. The relation of lymphocytes change and radiation pneumonitis was also confirmed in mice model. Therefore, decrease in lymphocyte and CD4^+^ T lymphocyte was able to act as a predictor of radiation pneumonitis in patients who received thoracic radiotherapy.

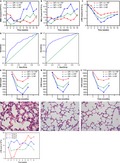

## 1 BACKGROUND

Radiotherapy (RT) is a major therapeutic strategy for patients with lung cancer. Radiation pneumonitis (RP) is the most common complication of RT for lung cancer and became a major reason that limited the utility of RT for patients with lung cancer.^1^, ^2^, ^3^, ^4^ Although numerous studies have sought to identify biological predictors of RP, but have been hampered for several reasons including difficulty with data analysis due to the release of many cytokines and growth factors by tumors in response to RT.^5^, ^6^, ^7^, ^8^, ^9^ Several predicting factor for RP found by previous studies were not satisfaction and feasibility to clinical application. The mechanisms of RP are complex and an accepted explanation for RP had largely centered on hypersensitivity reaction.^10^ On the pathophysiology level, a well‐defined RP has been suggested as a severe general immunoreaction, wherein inflammatory cells infiltrated into the pulmonary interstitium.^11^ Some evidence from preclinical and clinical investigations supports that recruitment or local infiltration of T lymphocytes had a causal connection with the complex inflammation course of RP and became a common characteristic of RP.^12^, ^13^, ^14^ Significantly increased lymphocytes in the bilateral bronchoalveolar lavage fluid of lung cancer patients were more pronounced in symptomatic patients than in asymptomatic ones.^15^ More importantly, detection of T lymphocyte subsets undertaken by flow cytometric analysis found that CD4^+^ T lymphocytes remarkably accumulated during radioactive pneumonia period in patients with lung cancer.^15^, ^16^

Thus, we hypothesized that variations in peripheral blood lymphocytes and T lymphocytes, which are routinely measured in clinic visits, may be associated with pneumonitis development induced by irradiation and should be considered predictors of RP. This investigation of dynamic variations in circulating lymphocytes and T lymphocyte subsets during RT and the RP period in lung cancer patients and experimental mice could help solve this problem.

## 2 METHODS

### 2.1 Patients

From January 2015 to December 2017, a total of 643 patients diagnosed with lung cancer in our institute according to the American Joint Committee on Cancer staging system, 6th edition, received definitive intensity‐modulated radiation therapy (IMRT). Patients (a) who were not undergoing a peripheral blood examination and a chest computed tomography (CT) scan according to requirements; (b) who have incomplete RT; (c) who were lost to follow‐up; (d) who have severe pulmonary bacterial infection since the start of this study; or (e) whose chemotherapy resulted in myelosuppression of WHO grade > 1, or treating with a tyrosine kinase inhibitor were excluded. Hence, 182 patients were eligible for analysis (128 non‐small cell lung cancer and 54 small cell lung cancer). The baseline characteristics, including age, gender, Karnofsky performance status (KPS), TNM staging of tumor, chemotherapy strategy of these patients, dose parameters, are displayed in Table 1. This study was approved by the appropriate ethics review board of Xinqiao Hospital and all methods used were performed in accordance with relevant guidelines and regulations. All study participants wrote and provided informed consent.

**TABLE 1:** Demographic and baseline clinical characteristics of patients

Variables	No. patients (N = 182)
Sex	
Male	148 (81.3%)
Female	34 (18.7%)
Age	
Median (range)	58 (34‐81)
<65 y	147 (80.8%)
≥65 y	35 (19.2%)
Tumor histology	
Adenocarcinoma	54 (29.7%)
Squamous	74 (40.7%)
Small cell carcinoma	54 (29.7%)
Clinical stage	
II	36 (19.8%)
III	72 (39.6%)
IV	74 (40.7%)
Concurrent chemotherapy	
Yes	89 (51.1%)
No	93 (48.9%)
KPS scores	
90	34 (18.7%)
80	129 (70.9%)
70	19 (10.4%)
Smoking status	
Smoker	127 (30.2%)
Nonsmoker	55 (69.8%)
Radiation pneumonitis	
0‐I	48 (26.4%)
II	48 (26.4%)
III	86 (47.3%)

### 2.2 Treatment regimen and delivery of IMRT, detection of peripheral blood

Gross tumor volume (GTV) and Gross tumor volume of metastatic lymph node (GTVnd) included primary tumor and involved lymph nodes, respectively. Clinical target volume (CTV) was determined of GTV and GTVnd plusing a 0.6‐0.8 cm margin and planning target volume (PTV) formed of CTV plusing 0.5 cm margin. The total dose of IMRT ranged from 54 to 60 Gy (median, 56 Gy), with a median treatment duration of 40 days, given by conventional fractionated radiation (1.8 or 2 Gy/fraction, 5 d/wk). The regimens of IMRT were made of the treatment planning system of EclipseTM version 8.0 or Oncentra MasterPlan 3.3. Treatment plans were evaluated with dose‐volume histogram and were considered acceptable if the PTVs were covered by 95% of the isodose curves, and dosage of normal organs was lower to their tolerances. Normal tissue dose‐volume constraints of this study were based on constraints of organ at risk in RTOG 0617 data. The dosage of RT was delivered with 6 MV X‐ray by linear accelerator (Trilogy, Varian Medical System Inc or Synergy, Elekta Precision Radiation Medicine).

White blood cell (WBC) measurements of all patients were performed in peripheral blood samples using standard phlebotomy techniques at four time points: at baseline (within 2 weeks prior to irradiation), at an interval of 2 weeks during RT, at least once from 2 weeks to 3 months after RT, and once every 1 or 2 weeks during the RP period if it occurred. The T lymphocyte subsets (CD4^+^, CD8^+^, and CD3^+^ T lymphocytes) of 129 patients were measured at the same time points.

### 2.3 Diagnosis and grading of radiation‐induced pneumonitis

Diagnosis of RP was based on combination of clinical manifestation, radiological images, and arterial blood gas index including dry cough, shortness of breath, radiologic findings on CT scan, and hypoxemia. The values of C‐reactive protein and procalcitonin were minor index of diagnosis. Gram staining and bacterial culture of sputum were performed to detect the bacterial infections of lungs if increasing of C‐reactive protein or procalcitonin in patient's blood plasma implied bacterial infection. Patients with severe pulmonary bacterial infection would be excluded. A multiple disciplinary team including two doctors of radiology, a doctor of radiotherapy, and a doctor of infectious disease agreed with the diagnosis of RP in patients of this study. RP was graded from 0 to 5 according to the CTCAE morbidity scoring classification.^17^

### 2.4 Follow‐up and toxicity evaluation

The follow‐up duration was defined as the time from the start date of RT to the last date of follow‐up for surviving patients or to the date of death. Patients underwent weekly interviews and symptom assessment; physical examination during the RT period; and a chest CT scan 1 month after completion of RT, every 2‐3 months during the first 2 years, and every 6 months thereafter for the third year.

### 2.5 Animal model and treatment protocol

Eight‐week‐old C57BL/6 female specific pathogen‐free healthy mice, weighing 15‐20 g（from Beijing Vital River Laboratory Animal Technology Co., Ltd.) were randomly divided into three groups; Group A: mice treated with 15 Gy of thoracic irradiation (N = 21), Group B: mice treated with 5 Gy thoracic radiation (N = 7), Group C: controls (N = 8). RT was simulated by Simulix HQ (Nucletron Tech) and was planned by EclipseTM version 8.0. Before irradiation, mice, anesthetized with intraperitoneal injection of pentobarbital, were fixed on the mice plate and the extremities, abdomen, and head were shielded with lead strips. Irradiation to lungs of mice was delivered with 6MeV‐electron rays by Trilogy. All mice of this study were housed three to five per cage and were breed in specific pathogen‐free housing systems under controlled temperature and cycle light/dark with free access to food and water. The prescription of irradiation for mice RP according to the project of reported study.^18^

The experimental mice were followed once a day and peripheral blood samples of mice were obtained from tail veins before radiation and once a week from 1 day to 49 days after irradiation. Blood cells were detected by Blood Cells Autoanalyzer according to the manufacturers’ instructions. All the mice experiments were approved by the Institutional Animal Care and Use Committee of Xinqiao Hospital and all methods were performed in accordance with the relevant guidelines and regulations.

### 2.6 Histological analysis of experimental animals

The experimental mice were executed after anesthetized with intraperitoneal injection of pentobarbital at 8 weeks after radiation. The time point of executing mice for collecting the RP tissue was according to Zhou's study of radiation‐induced lung injury in mouse model.^19^ Lung tissues of mice were obtained, fixed in 10% formalin, and embedded in paraffin. Tissue sections were prepared for histological analysis with hematoxylin and eosin (H&E) stain. Analysis of RP was performed by a doctor of pathology.

### 2.7 Statistical methods

To compare the difference in blood cell measurements over time, the test group was divided according to successive 2‐week intervals and stratified by the grade of RP. The one‐way ANOVA was used to compare the difference in lymphocyte counts among three subgroups based on RP classification. Univariate and multivariate analyses were calculated via logistic regression. To create a multivariate model, we used logistic stepwise regression with variables entry at *P* < .15 and removed if the P value was >0.15. Recursive partitioning analysis was used to calculate the best cutoff value for successive variables for grade 2 and 3 RP via JMP (version 10; SAS Institute). Receiver operating characteristic (ROC) curve analysis was adopted to explore the discriminative accuracy of lymphocyte count in distinguishing patients with grade 2‐3 RP and without RP. The area under the curve (AUC) was used to summarize the predictive ability and standard errors; 95% confidence intervals (CIs) for the AUC values were constructed by nonparametric bootstrapping. JMP software program and SPSS (version 17.0; SPSS Inc) were used for statistical analyses; *P*‐values are from two‐sided tests. *P* < .05 was considered statistically significant.

## 3 RESULTS

During a median follow‐up time of 16.5 months (range, 3‐44 months), symptomatic and severe RP occurred at a median of 6.4 weeks (range, 2‐24 weeks) after the end of RT, equivalent to 12 weeks after irradiation start. We observed grade 0‐1 RP in 48 patients (26.4%), grade 2 RP in 48 patients (26.4%), and grade 3 RP in 86 patients (47.3%).

### 3.1 WBC, neutrophil, and lymphocyte counts of patients with radiotherapy to lung cancer

**FIGURE 1:**
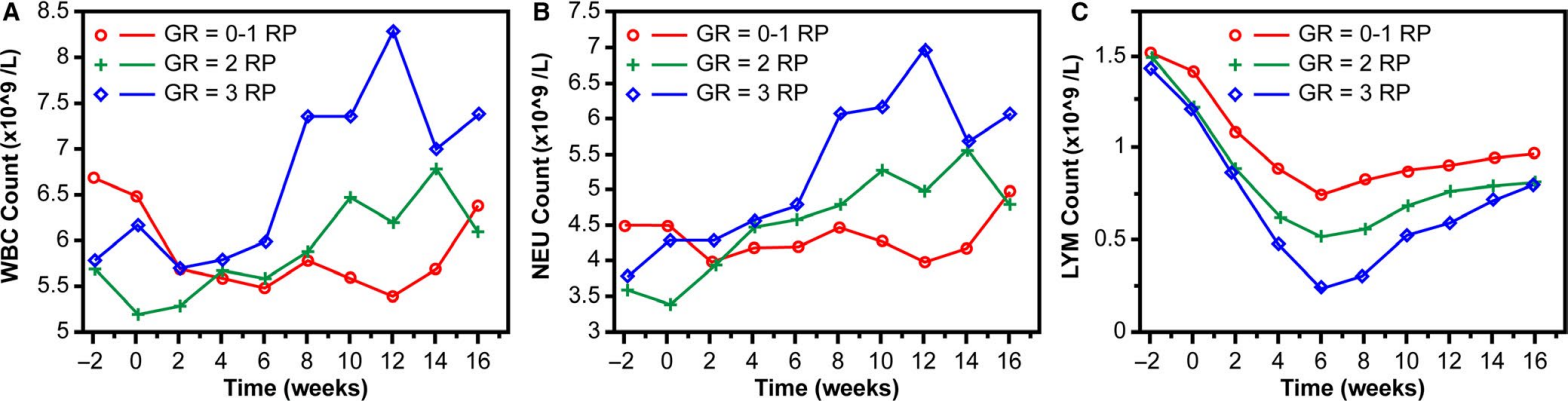
Mean blood cell counts over time for patients with RP. The mean of blood cell counts in patients with grade (GR) 0‐1, GR 2, or GR 3 RP were shown as red, green, and blue curve, respectively. The x‐axis represents time, and the ordinate shows the mean blood cell counts. Blood cell measurements are plotted within successive 2‐wk intervals, time 0 indicates RT start date, and negative values indicate time prior to RT. A: WBC count; B: neutrophil (NEU) count; C: lymphocyte count

No significant correlation was detected between RP and acidophil cells, basophil cells, and monocytes. A summary of WBC, neutrophil, and lymphocyte counts evaluated at 2‐week intervals prior to, during, and after RT was displayed, and to visualize blood cell trends over time, these numerical values were plotted according to the time at which RT begins. There was no statistically significant difference in the absolute levels of WBCs and neutrophils between patients who experienced grade 0‐1 RP and grade 2 RP, but we found a sharp increase in WBCs and neutrophils at week 12 postirradiation in patients who experienced grade 3 RP (Table S1; Figure S1A,B). Lymphocyte levels decreased markedly in patients who developed grade 2 or 3 RP compared with patients with grade 0 or 1 RP from the second to the sixth week of the RT start, and recovered gradually after 2 weeks of thoracic irradiation end (Figure 1C). Furthermore, more severe RP was associated with lower levels of lymphocytes during the time window of RP development. Mean lymphocyte numbers were 0.46 × 10^9^/L for those with grade 3 RP, 0.65 × 10^9^/L for those with grade 2 RP, and 0.89 × 10^9^/L for those with grade ≤ 1 RP. Differences in mean lymphocyte counts were significant for grade 3 vs grade 2 (*P* < .001) and grade ≤ 1 RP (*P* < .001) (Figure S1).

### 3.2 Predictive risk assessment results of univariate and multivariate analyses

Utilizing recursive partitioning analysis, we found threshold values of lymphocytes demarcating grade 2 RP to be 0.59 and grade 3 RP to be 0.37. To predict the risk of RP, univariate and multivariate analyses were performed. On univariate analysis, lymphocyte count was a significant factor for RP (*P* < .005). Moreover, the results of multivariable analysis showed that lymphocyte count can be an independent prognostic factor for RP (*P* < .005) (Table S2).

**FIGURE 2:**
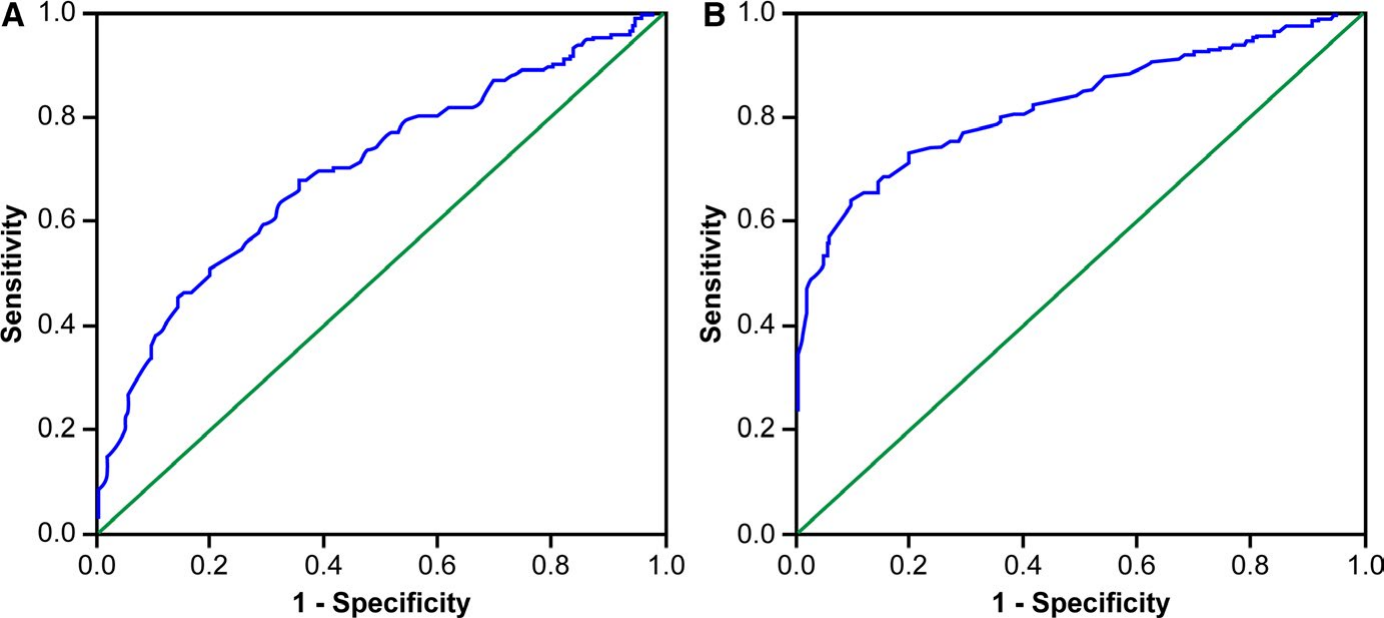
ROC curves based on the sensitivity and specificity of lymphocytes. A: lymphocytes for grade 2 RP (AUC = 0.689); B: lymphocytes for grade 3 RP (AUC = 0.824)

To evaluate the accuracy of lymphocyte count as a biomarker for predicting RP, we performed ROC curve analysis, which revealed that the AUC values of lymphocytes were higher than the line of no discrimination (defined as AUC Z.50) for both grade 2 and 3 RP. For grade 2 RP, the AUC was 0.698, and for grade 3 RP, it was 0.824 (Figure 2).

### 3.3 The results of T lymphocyte subsets in patients with RP

**FIGURE 3:**
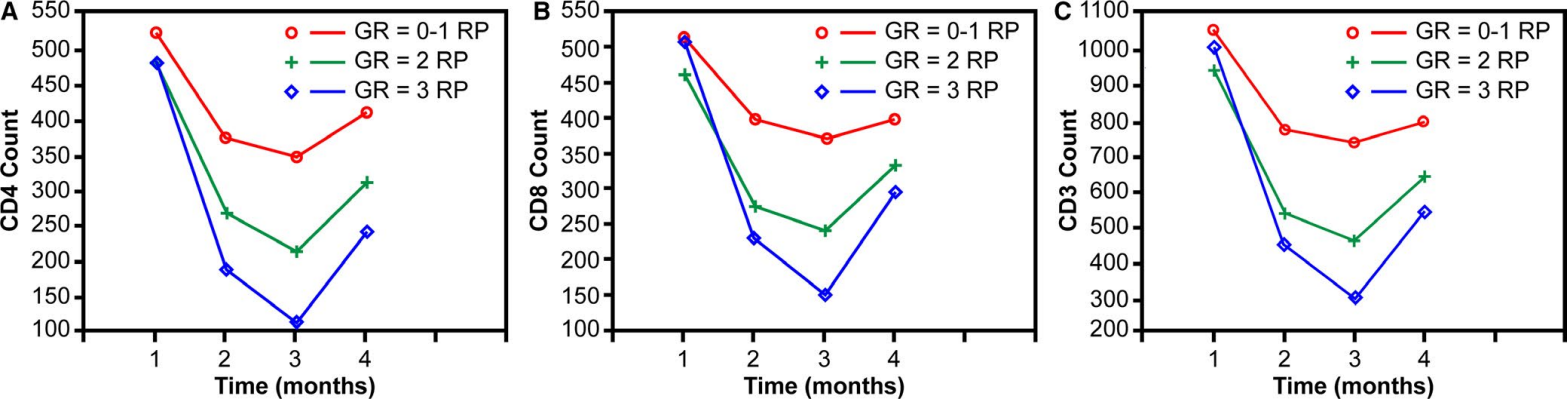
Mean counts of T lymphocyte subsets over time for patients with RP. The red, green, and blue curve represented the variance of T‐cell subsets in patients with grade (GR) 0‐1, grade 2, or grade 3 RP, respectively. The x‐axis represents time. T lymphocyte measurements are plotted within successive 1‐month intervals, and time 0 indicates RT start date. A: CD4^+^ count; B: CD8^+^ count; C: CD3^+^ count

The baseline clinical characteristics of 129 patients included in this study are listed in Table S3. The results showed that levels of T lymphocyte subsets all decreased when RP occurred, and increased during the recovery period of RP, and the lowest counting of T lymphocyte subsets occurred on the third month of RT start (Figure 3); levels of CD4^+^ T lymphocytes were significantly lower in patients with grade 2 or 3 RP than in patients with grade 0‐1 RP (*P* < .05) (Table S4). Moreover, there was a significant inverse relation between the grade of RP and the number of CD4^+^ T lymphocytes (Figure S2). On univariate and multivariate analyses, we found that CD4^+^ T lymphocyte was statistically significant as an independent predictive factor for RP (*P* < .001) (Table S5).

### 3.4 Lymphocyte counts and RP analysis of animal model

**FIGURE 4:**
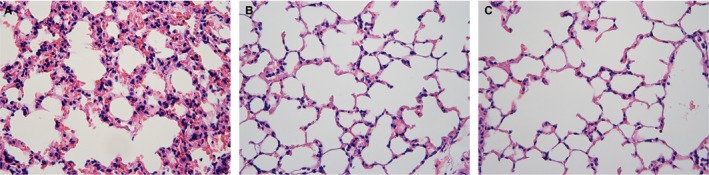
HE staining on lung tissue in mice received thoracic irradiation with different dose. A, There is an accumulation of numerous inflammatory cells in alveolar spaces, intra‐alveolar hyaline membrane formation, thickness of bronchiolar epithelium, and fibrotic alveolar septum. B and C, There was no radiation‐induced lung injury in mice

It was difficult to design a prospective clinical trial for exploring the predictor of RP. Therefore, experimental mice model of RP was used to confirm the previous results of patients with RP in this study. All 36 mice fulfilled this study. Radiation‐induced lung damage occurred in the lungs of group A mice. It showed accumulation of numerous inflammatory cells in alveolar spaces, intra‐alveolar hyaline membrane formation, thickness of bronchiolar epithelium, and fibrotic alveolar septum (Figure 4A). There was no radiation‐induced lung injury in mice of groups B and C (Figure 4B,C).

**FIGURE 5:**
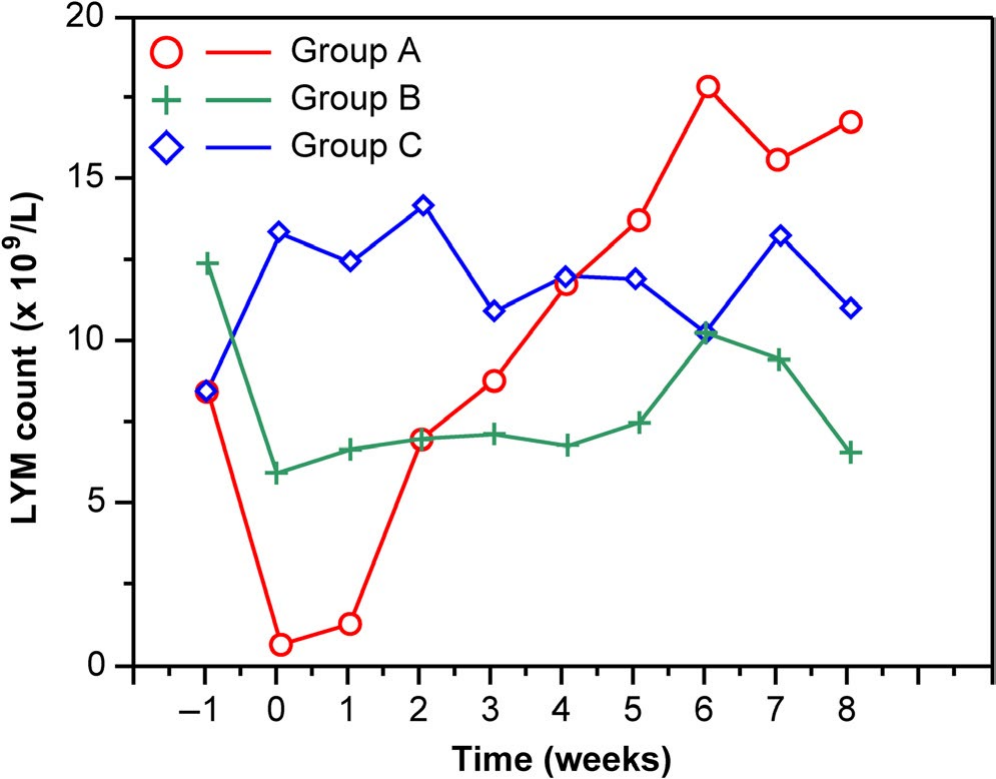
Mean lymphocyte counts over time for mice with group A, B, and C. The x‐axis represents time, and the ordinate shows the lymphocyte counts. Blood cell measurements are plotted within successive 1‐week intervals, time 0 indicates RT start date, and negative values indicate time prior to RT

The lymphocyte levels decreased quickly after radiation in mice of groups A and B compared to group C and recovered at 3 weeks after radiation. The lymphocyte counts of group A decreased markedly than groups B and C (*P* < .001). The lowest value of lymphocytes was 0.8 × 10^9^/L in group A compared to 6.1 × 10^9^/L in group B at the second day postirradiation (Figure 5). The changes of lymphocytes level in mice received radiation to lungs were similar to patients with RT to lung cancer. The decreasing of lymphocytes level was linked closely with RP occurrence and was associated with the RP in experimental animal model based on the histological analysis of radiated lungs in mice.

## 4 DISCUSSION

Our study identified that reduced levels of lymphocytes were significantly correlated with the occurrence of RP induced by irradiation in patients with lung cancer. Particularly, we found that a meaningful drop in circulating lymphocyte count occurred at 6 weeks and cutoff values of lymphocytes demarcating grade 2 RP to be 0.59 and grade 3 RP to be 0.37. Grade 2 to 4 RP often interrupted the therapeutic strategy of patients with lung cancer and serious RP may result in death of patients who received thoracic RT. It is important to find out the patients before they have obvious symptom of RP. The results of univariate and multivariate analyses both showed that lymphocyte count and CD4^+^ T lymphocyte may be independent prognostic factors for RP. The observed drop in lymphocyte count and T lymphocyte subsets before the occurring symptom of RP in patients with thoracic radiation may provide an early warning for clinical management. In experimental animal model, the increasing of neutrophils rate and decreasing of lymphocytes level occurred in peripheral blood of mice after thorax irradiation. Moreover, the decreasing of lymphocytes level was better correlated with the occurrence of RP than the neutrophils rate. The change of lymphocytes level during the radiation‐induced lung damage in mice nicely reproduces the pathogenesis and time course of RP in patients. Moreover, the severity of RP was associated with the degree of lymphocyte decrease. Utilizing ROC curve analysis, the accuracy of lymphocyte counts associated with RP was found to be reliable, especially for grade 3 RP, which is more specific and sensitive than grade 2 RP. This biomarker could be cost‐effective and readily available to aid in RP diagnosis and help prescribe a personalized therapeutic strategy. Typically, some patients with grade 0/1 RP and grade 4/5 were not included in this study because of no regular peripheral blood examination/chest CT scanning or combining with severe pulmonary infection. It resulted in selection bias that might have impact on the results of lymphocyte and T lymphocyte subsets analysis.

In addition to inducing direct DNA damage of cells, irradiation can stimulate immune cells and promote immune response.^20^, ^21^ Several evidences suggested that the RT‐mediated hypersensitivity reaction was caused by a severe lymphoid interstitial pneumonitis.^22^, ^23^ The radiation‐induced lungs injury observed in experimental mice showed accumulation of numerous inflammatory cells in alveolar septum, intra‐alveolar hyaline membrane formation, thickness of bronchiolar epithelium, and fibrotic alveolar septum. RP often exhibits a distinctive feature: an “out‐of‐field” response to localized lung irradiation, the same lymphocytosis of lavage in both irradiated and unirradiated lung segments. Most of the cell mixture in the bronchoalveolar lavage of “normal” lung interstitium comprised lymphocytes, which accounted for 60%‐70% of the total counts in patients with RP.^15^, ^16^, ^22^ Another character of RP is its unpredictable and sporadic onset.^24^ The pathological inflammation outside the area of irradiation identified by a gallium scan of the lung and bilateral bronchoalveolar lavage implied that it was the mobilization of the cytokine cascade. Numerous observations had detected lymphocytes and T lymphocyte subsets as key mediators in RP. A series of lung biopsies demonstrated that lymphocytic infiltration was a dominant finding in the irradiated lungs with acute RP.^24^ All these evidence implied that decrease in circulating lymphocytes could be due to lymphocytes infiltrating into the lung interstitium and reasonably explains the decrease in circulating lymphocytes during pre‐RP and RP, and the recovery along with the remission of RP in patients included in our study.

Besides, change of lymphocyte in this study, the count of T lymphocyte subsets shows a distinctive fluctuation after radiation in patients with RP. T cells contribute to radiation‐induced lung injury and thoracic irradiation significantly affected the numbers of T cells in lungs and the bronchoalveolar lavage.^25^, ^26^ A detailed analysis of bronchoalveolar lavage showed a dramatical increase of lymphocytes, mainly including the CD4^+^ T lymphocytes, in breast cancer patients with RP after irradiation.^3^ Wirsdörfer et al detected T‐cell compartments from lung tissue, spleen, and cervical lymph nodes in mice with thoracic RT. They found that irradiation triggered dual effects of immune system: local immunoactivation and systemic immunosuppression. It provoked an increased influx of CD3^+^ T cells at 6 and 12 weeks postirradiation in lung tissue accompanied by reduction of CD3^+^ T cells in peripheral lymphoid tissues. In detail, CD4^+^ T cell count was transiently reduced while CD8^+^ T cell count sustained a decrease in peripheral lymphoid organs.^14^ Thus, the change of T lymphocyte subsets in peripheral blood may be reflected indirectly in its enrichment to lungs and participating process of inflammation. It might act as an independent predictive factor for RP, as confirmed in our study.

Early detection of radioactive pneumonia is increasingly important with the results of the Pacific study.^27^ Variations in lymphocytes, including T lymphocytes subsets, were found to be important factors in the RP inflammation process and were associated with RP occurrence. This relationship could potentially establish a rapid diagnostic and predictive assay of each patient's risk profile. The peripheral blood analysis might be a good option for providing a warning of irradiation‐induced lung injury. Tang et al demonstrated that patients with grade 3 RP had higher percentages of WBCs than patients without RP.^28^ A similar trend was observed in our research. Moreover, our study found that lymphocyte counts had already decreased before RP symptoms developed, especially with low‐grade RP. This can be important for medical treatment, offering a chance for an early intervention to prevent RP exacerbation.

## 5 CONCLUSIONS

In this study, we analyzed the dynamic variations in the levels of lymphocytes and T lymphocyte subsets in patients who received RT for lung cancer. We found that lymphocyte count decreased markedly in patients who developed grade 2 or 3 RP compared with patients with grade 0‐1 RP, and more severe RP was associated with lower levels of lymphocytes during the time window of RP development. The decreasing of lymphocytes level was also confirmed in radiation‐induced lung damage of experimental mice. CD4^+^ T cells were also statistically associated with RP. The levels of lymphocytes and T lymphocyte subsets in peripheral blood can be a simple and useful indicators for screening patients who may show an increased probability of radiation‐induced lung toxicity. Further, our results may aid clinicians in RP risk assessment. Certainly, this study was a retrospective analysis; a prospective observational trial would be ideal to confirm our results, and further studies are required to explore the mechanism of lymphocytes participating in RP.

## CONFLICT INTERESTS

None declared.

## AUTHOR CONTRIBUTIONS

PZ and GL were involved in conception, design, and writing of this study. LC was involved in analysis of data. DY, CC, and SZ were involved in Diagnosis of RP; CH and DC were involved in work of experimental animal; PZ, GC, ZW, LZ, and WL were involved in the collection and assembly of patients’ data.

## Supporting information

Table S1‐S5

Fig S1‐S2

## Data Availability

All data generated or analyzed during this study are shown in this published article and supplementary material.
