# Supplementary material for: Early variations in lymphocytes and T lymphocyte subsets are associated with radiation pneumonitis in lung cancer patients and experimental mice received thoracic irradiation
Source: Cancer Med. 2020 Mar 24;9(10):3437–44. doi: 10.1002/cam4.2987 (PMC7221303; doi:10.1002/cam4.2987)
Supplement: Supplementary file 1 — Table S1‐S5 [file CAM4-9-3437-s001.doc]

**Supplementary Table 1.** Correlation between blood cells and RP.

| **Blood cells** | **GR 0-1 RP** | | **GR 2 RP** | | **GR 3 RP** | | ***P* value*** | | |
| --- | --- | --- | --- | --- | --- | --- | --- | --- | --- |
|  | **Mean** | **SD** | **Mean** | **SD** | **Mean** | **SD** | **GR 0-1**  **vs. GR 2** | **GR 0-1 vs. GR3** | **GR 2**  **vs. GR3** |
| WBCs (10^9^/L) |  |  |  |  |  |  |  |  |  |
| W -2 | 6.7 | 2.6 | 5.7 | 2.3 | 5.8 | 2.3 | 0.103 | 0.169 | 1.000 |
| W 0 | 6.5 | 2.8 | 5.2 | 1.7 | 6.2 | 2.6 | 0.032 | 1.000 | 0.057 |
| W 2 | 5.7 | 2.6 | 5.3 | 2.5 | 5.7 | 3.0 | 1.000 | 1.000 | 1.000 |
| W 4 | 5.6 | 2.6 | 5.7 | 2.8 | 5.8 | 3.2 | 1.000 | 1.000 | 1.000 |
| W 6 | 5.5 | 2.4 | 5.6 | 2.9 | 6.0 | 2.6 | 1.000 | 0.642 | 1.000 |
| W 8 | 5.8 | 2.6 | 5.9 | 2.0 | 7.4 | 3.8 | 1.000 | 0.016 | 0.029 |
| W 10 | 5.6 | 2.2 | 6.5 | 2.9 | 7.4 | 3.4 | 0.476 | 0.006 | 0.362 |
| W 12 | 5.4 | 2.0 | 6.2 | 3.2 | 8.3 | 3.5 | 0.773 | 0.000 | 0.001 |
| W 14 | 5.7 | 1.8 | 6.8 | 3.2 | 7.0 | 3.1 | 0.221 | 0.053 | 1.000 |
| W 16 | 6.4 | 2.9 | 6.1 | 2.2 | 7.4 | 3.4 | 1.000 | 0.154 | 0.044 |
| NEUT (10^9^/L) |  |  |  |  |  |  |  |  |  |
| W -2 | 4.5 | 2.5 | 3.6 | 2.2 | 3.8 | 2.2 | 0.149 | 0.270 | 1.000 |
| W 0 | 4.5 | 2.3 | 3.4 | 1.4 | 4.3 | 2.6 | 0.072 | 1.000 | 0.078 |
| W 2 | 4.0 | 2.5 | 3.9 | 2.5 | 4.3 | 2.9 | 1.000 | 1.000 | 1.000 |
| W 4 | 4.2 | 2.1 | 4.5 | 2.6 | 4.6 | 3.2 | 1.000 | 1.000 | 1.000 |
| W 6 | 4.2 | 2.3 | 4.6 | 2.8 | 4.8 | 2.6 | 1.000 | 0.566 | 1.000 |
| W 8 | 4.5 | 2.5 | 4.8 | 2.0 | 6.1 | 3.9 | 1.000 | 0.017 | 0.064 |
| W 10 | 4.3 | 2.0 | 5.3 | 3.1 | 6.2 | 3.5 | 0.301 | 0.002 | 0.291 |
| W 12 | 4.0 | 2.1 | 5.0 | 3.2 | 7.0 | 3.7 | 0.386 | 0.000 | 0.002 |
| W 14 | 4.2 | 1.7 | 5.6 | 3.2 | 5.7 | 3.2 | 0.074 | 0.018 | 1.000 |
| W 16 | 5.0 | 2.8 | 4.8 | 2.3 | 6.1 | 3.3 | 1.000 | 0.141 | 0.069 |
| LYM (10^9^/L) |  |  |  |  |  |  |  |  |  |
| W -2 | 1.5 | 0.5 | 1.5 | 0.4 | 1.4 | 0.5 | 1.000 | 0.851 | 1.000 |
| W 0 | 1.4 | 0.6 | 1.2 | 0.4 | 1.2 | 0.4 | 0.125 | 0.030 | 1.000 |
| W 2 | 1.1 | 0.5 | 0.9 | 0.4 | 0.8 | 0.3 | 0.042 | 0.001 | 0.999 |
| W 4 | 0.9 | 0.5 | 0.6 | 0.3 | 0.5 | 0.2 | 0.000 | 0.000 | 0.040 |
| W 6 | 0.8 | 0.3 | 0.5 | 0.2 | 0.2 | 0.1 | 0.000 | 0.000 | 0.000 |
| W 8 | 0.8 | 0.4 | 0.6 | 0.3 | 0.3 | 0.2 | 0.000 | 0.000 | 0.000 |
| W 10 | 0.9 | 0.4 | 0.7 | 0.3 | 0.5 | 0.2 | 0.010 | 0.000 | 0.012 |
| W 12 | 0.9 | 0.3 | 0.8 | 0.3 | 0.6 | 0.3 | 0.096 | 0.000 | 0.003 |
| W 14 | 1.0 | 0.4 | 0.8 | 0.3 | 0.7 | 0.3 | 0.098 | 0.001 | 0.553 |
| W 16 | 1.0 | 0.3 | 0.8 | 0.3 | 0.8 | 0.4 | 0.156 | 0.048 | 1.000 |

* Multiple-sample comparison of the mean, one-way ANOVA with Bonferroni correction, performed on log2 scale.

Abbreviations: GR, grade; SD, standard deviation; W, week; NEUT, neutrophils; LYM, lymphocytes.

**Supplementary Table 2.** Stepwise univariate and multivariate logistic regression analyses.

| **Variables** | UVA ( RP grade 2)  UVA | | | MVA ( RP grade 2)  MUV | | | UVA ( RP grade 3)  UVA | | | MVA ( RP grade 3)  MVA | | |
| --- | --- | --- | --- | --- | --- | --- | --- | --- | --- | --- | --- | --- |
|  | OR | 95%CI | ***P*** | OR | 95%CI | ***P*** | OR | 95%CI | ***P*** | OR | 95%CI | ***P*** |
| Sex Male | 1.1 | 0.4-2.9 | 0.805 | NI |  |  | 0.6 | 0.2-1.6 | 0.402 | NI |  |  |
| Female | 0^a^ |  |  |  |  |  | 0^a^ |  |  |  |  |  |
| Age ≤65 years | 0.4 | 0.1-1.4 | 0.187 | NI |  |  | 0.5 | 0.2-1.4 | 0.227 | NI |  |  |
| >65 years | 0^a^ |  |  |  |  |  | 0^a^ |  |  |  |  |  |
| Tumor histology |  |  |  | NI |  |  |  |  |  | NI |  |  |
| adenocarcinoma | 0.5 | 0.2-1.3 | 0.163 |  |  |  | 1.2 | 0.5-2.9 | 0.723 |  |  |  |
| Squamous | 0.7 | 0.3-1.7 | 0.394 |  |  |  | 1.5 | 0.6-3.7 | 0.355 |  |  |  |
| Small cell carcinoma | 0^a^ |  |  |  |  |  | 0^a^ |  |  |  |  |  |
| Clinical stage II | 0.4 | 0.1-1.1 | 0.098 | NI |  |  | 0.9 | 0.4-2.4 | 0.914 | NI |  |  |
| III | 0.8 | 0.3-1.9 | 0.550 |  |  |  | 1.4 | 0.6-3.1 | 0.401 |  |  |  |
| IV | 0^a^ |  |  |  |  |  | 0^a^ |  |  |  |  |  |
| Chemotherapy |  |  |  | NI |  |  |  |  |  | NI |  |  |
| No | 1.8 | 0.6-5.7 | 0.295 |  |  |  | 0.9 | 0.4-2.3 | 0.893 |  |  |  |
| Concurrent platinum | 2.7 | 0.8-9.4 | 0.126 |  |  |  | 1.6 | 0.6-4.3 | 0.398 |  |  |  |
| Concurrent taxane | 0^a^ |  |  |  |  |  | 0^a^ |  |  |  |  |  |
| KPS scores 70 | 0.8 | 0.2-3.2 | 0.715 | 1.4 | 0.3-6.8 | 0.652 | 0.2 | 0.1-0.8 | 0.025 | 0.5 | 0.1-2.8 | 0.405 |
| 80 | 0.9 | 0.3-2.7 | 0.816 | 1.6 | 0.5-5.4 | 0.462 | 0.7 | 0.3-1.8 | 0.474 | 1.9 | 0.6-6.5 | 0.291 |
| 90 | 0^a^ |  |  | 0^a^ |  |  | 0^a^ |  |  | 0^a^ |  |  |
| Smoking status |  |  |  | NI |  |  |  |  |  | NI |  |  |
| Nonsmoker | 0.8 | 0.3-1.8 | 0.513 |  |  |  | 0.7 | 0.3-1.5 | 0.367 |  |  |  |
| Smoker | 0^a^ |  |  |  |  |  | 0^a^ |  |  |  |  |  |
| LYM count W6 |  |  |  |  |  |  |  |  |  |  |  |  |
| <0.37 | 8.5 | 2.1-35.2 | 0.003 | 9.6 | 2.2-41.6 | 0.002 | 52.77 | 13.7-203.55 | 0.000 | 60.6 | 14.7-249.5 | 0.000 |
| ≤0.59 and ≥0.37 | 3.1 | 1.3-7.8 | 0.014 | 3.4 | 1.3-8.6 | 0.011 | 3.1 | 1.2-8.2 | 0.024 | 3.2 | 1.2-8.9 | 0.023 |
| >0.59 | 0^a^ |  |  | 0^a^ |  |  | 0^a^ |  | 0^a^ |  |  |  |
| Dose Parameters |  |  |  |  |  |  |  |  |  |  |  |  |
| Lung V5 (%) |  |  |  | NI |  |  |  |  |  | NI |  |  |
| ＜50.9% | 1.0 | 0.4-2.2 | 1.000 |  |  |  | 0.6 | 0.3-1.3 | 0.182 |  |  |  |
| ≥50.9% | 0^a^ |  |  |  |  |  | 0^a^ |  |  |  |  |  |
| Lung V20 (%) |  |  |  | NI |  |  |  |  |  | NI |  |  |
| ＜25.4% | 0.9 | 0.4-2.1 | 0.837 |  |  |  | 0.6 | 0.3-1.1 | 0.111 |  |  |  |
| ≥25.4% | 0^a^ |  |  |  |  |  | 0^a^ |  |  |  |  |  |
| MLD (cGy) |  |  |  | NI |  |  |  |  |  | NI |  |  |
| ＜1300cGy | 0.9 | 0.4-2.0 | 0.838 |  |  |  | 0.5 | 0.3-1.1 | 0.097 |  |  |  |
| ≥1300cGy | 0^a^ |  |  |  |  |  | 0^a^ |  |  |  |  |  |
| GTV (cm^3^) |  |  |  | NI |  |  |  |  |  |  |  |  |
| ＜170.6cm^3^ | 1.0 | 0.4-2.2 | 1.0 |  |  |  | 0.8 | 0.4-1.7 | 0.625 |  |  |  |
| ≥170.6cm^3^ | 0^a^ |  |  |  |  |  | 0^a^ |  |  |  |  |  |

^a^ This parameter is set to zero.

Abbreviations: UVA, univariate analysis; MVA, multivariate analysis; OR, odds ratio; NI, not included in the multivariate regression model; LYM, lymphocytes; MLD, mean lung dose; GTV, gross tumor volume.

Characteristics were entered into the multivariate model in a stepwise fashion if *P*<0.15 and were removed at any point if *P* was >0.15.

**Supplementary Table 3. Demographic and baseline clinical characteristics of patients.**

| **Variables** | **No. of patients (N = 129)** |
| --- | --- |
| Sex |  |
| Male | 100 (77.5%) |
| Female | 29 (22.5%) |
| Age |  |
| Median (range) | 56 (34-80) |
| ≤65 years | 108 (80.8%) |
| >65 years | 21 (19.2%) |
| Tumor histology |  |
| Adenocarcinoma | 45 (34.9%) |
| Squamous | 48 (37.2%) |
| Small cell carcinoma | 36 (27.9%) |
| Clinical stage |  |
| II | 28 (21.7%) |
| III | 47 (36.4%) |
| IV | 54 (41.9%) |
| Concurrent chemotherapy |  |
| No | 65 (50.4%) |
| Concurrent platinum | 40 (31.0%) |
| Concurrent taxane | 40 (18.6%) |
| KPS scores |  |
| 90 | 24 (18.6%) |
| 80 | 94 (72.9%) |
| 70 | 11 (8.5%) |
| Smoking status |  |
| Smoker | 86 (66.7%) |
| Nonsmoker | 43 (33.3%) |
| Radiation pneumonitis |  |
| 0-I | 34 (26.4%) |
| II | 39 (30.2%) |
| III | 56 (43.4%) |
| Dose Parameters |  |
| Lung V5 (%) | 51.6 (15.2-87.9) |
| Lung V20 (%) | 25.4 (2.6-35.7) |
| MLD (cGy) | 1384 (306-2130) |
| GTV (cm^3^) | 178.1 (12.1-1102.7) |

**Supplementary Table 4. Correlation between T lymphocytes (T LYM) and RP**

| **T LYM** | **GR 0-1 RP** | | **GR 2 RP** | | **GR 3 RP** | | ***P* value*** | | |
| --- | --- | --- | --- | --- | --- | --- | --- | --- | --- |
|  | **Mean** | **SD** | **Mean** | **SD** | **Mean** | **SD** | **0-1 vs. 2** | **0-1 vs. 3** | **2 vs. 3** |
| CD4+ (/uL) |  |  |  |  |  |  |  |  |  |
| M 0 | 522 | 182 | 479 | 134 | 484 | 155 | 0.749 | 0.808 | 1.000 |
| M 1 | 372 | 114 | 266 | 99 | 185 | 82 | 0.000 | 0.000 | 0.000 |
| M 2 | 347 | 136 | 211 | 66 | 110 | 85 | 0.000 | 0.000 | 0.000 |
| M 3 | 410 | 175 | 310 | 85 | 240 | 104 | 0.002 | 0.000 | 0.022 |
| CD8+ (/uL) |  |  |  |  |  |  |  |  |  |
| M 0 | 513 | 135 | 461 | 164 | 504 | 165 | 0.485 | 1.000 | 0.597 |
| M 1 | 400 | 131 | 274 | 140 | 229 | 139 | 0.001 | 0.000 | 0.353 |
| M 2 | 370 | 139 | 240 | 113 | 149 | 96 | 0.000 | 0.000 | 0.001 |
| M 3 | 401 | 178 | 335 | 138 | 297 | 127 | 0.170 | 0.004 | 0.652 |
| CD3+ (/uL) |  |  |  |  |  |  |  |  |  |
| M 0 | 1056 | 324 | 943 | 264 | 1007 | 307 | 0.328 | 1.000 | 0.930 |
| M 1 | 778 | 243 | 541 | 207 | 450 | 208 | 0.000 | 0.000 | 0.138 |
| M 2 | 740 | 264 | 463 | 174 | 300 | 182 | 0.000 | 0.000 | 0.001 |
| M 3 | 797 | 335 | 643 | 200 | 549 | 198 | 0.023 | 0.000 | 0.197 |

* Multiple-sample comparison of the mean, one-way ANOVA with Bonferroni correction, performed on log2 scale.

Abbreviations: GD, grade; SD, standard deviation; M, month.

**Supplementary Table 5.** Stepwise univariate and multivariate logistic regression analyses.

| **Variables** | UVA ( RP grade 2)  UVA | | | MVA ( RP grade 2)  MUV | | | UVA ( RP grade 3)  UVA | | | MVA ( RP grade 2)  MVA | | |
| --- | --- | --- | --- | --- | --- | --- | --- | --- | --- | --- | --- | --- |
|  | OR | 95%CI | ***P*** | OR | 95%CI | ***P*** | OR | 95%CI | ***P*** | OR | 95%CI | ***P*** |
| Sex Male | 0.1 | 0.01-0.8 | 0.034 | 1.5 | 0.3-6.8 | 0.626 | 0.1 | 0.02-1.0 | 0.051 | 0.7 | 0.1-4.8 | 0.710 |
| Female | 0^a^ |  |  | 0^a^ |  |  | 0^a^ |  |  | 0^a^ |  |  |
| Age ≤65 years | 0.4 | 0.1-1.4 | 0.187 | NI |  |  | 0.5 | 0.2-1.4 | 0.227 | NI |  |  |
| >65 years | 0^a^ |  |  |  |  |  | 0^a^ |  |  |  |  |  |
| Tumor histology |  |  |  | NI |  |  |  |  |  | NI |  |  |
| adenocarcinoma | 0.6 | 0.2-1.9 | 0.403 |  |  |  | 0.8 | 0.3-2.3 | 0.769 |  |  |  |
| Squamous | 1.3 | 0.4-4.2 | 0.717 |  |  |  | 2.6 | 0.8-8.1 | 0.101 |  |  |  |
| Small cell carcinoma | 0^a^ |  |  |  |  |  | 0^a^ |  |  |  |  |  |
| Clinical stage II | 0.4 | 0.1-1.3 | 0.125 | NI |  |  | 0.9 | 0.3-2.5 | 0.793 | NI |  |  |
| III | 1.1 | 0.4-3.2 | 0.853 |  |  |  | 1.5 | 0.5-4.0 | 0.456 |  |  |  |
| IV | 0^a^ |  |  |  |  |  | 0^a^ |  |  |  |  |  |
| Chemotherapy |  |  |  | NI |  |  |  |  |  | NI |  |  |
| No | 1.2 | 0.3-4.2 | 0.826 |  |  |  | 0.6 | 0.2-1.7 | 0.583 |  |  |  |
| Concurrent platinum | 5.3 | 1.1-25.8 | 0.041 |  |  |  | 3.1 | 0.7-12.6 | 0.122 |  |  |  |
| Concurrent taxane | 0^a^ |  |  |  |  |  | 0^a^ |  |  |  |  |  |
| KPS scores 70 | 4.5 | 0.6-34.6 | 0.148 | NI |  |  | 0.6 | 0.1-4.9 | 0.669 | NI |  |  |
| 80 | 1.7 | 0.4-6.6 | 0.462 |  |  |  | 0.6 | 0.2-1.9 | 0.422 |  |  |  |
| 90 | 0^a^ |  |  |  |  |  | 0^a^ |  |  | 0^a^ |  |  |
| Smoking status |  |  |  | NI |  |  |  |  |  | NI |  |  |
| Nonsmoker | 0.9 | 0.3-2.3 | 0.837 |  |  |  | 0.6 | 0.3-1.6 | 0.343 |  |  |  |
| Smoker | 0^a^ |  |  |  |  |  | 0^a^ |  |  |  |  |  |
| Dose Parameters |  |  |  |  |  |  |  |  |  |  |  |  |
| Lung V5 (%) |  |  |  |  |  |  |  |  |  |  |  |  |
| ＜50.9% | 2.3 | 0.9-5.8 | 0.089 | 9.2 | 1.8-46.8 | 0.007 | 0.7 | 0.3-1.7 | 0.429 | 3.5 | 0.6-21.7 | 0.186 |
| ≥50.9% | 0^a^ |  |  |  |  |  | 0^a^ |  |  | 0^a^ |  |  |
| Lung V20 (%) |  |  |  | NI |  |  |  |  |  | NI |  |  |
| ＜25.4% | 1.2 | 0.5-3.0 | 0.719 |  |  |  | 0.7 | 0.3-1.7 | 0.470 |  |  |  |
| ≥25.4% | 0^a^ |  |  |  |  |  | 0^a^ |  |  |  |  |  |
| MLD (cGy) |  |  |  | NI |  |  |  |  |  | NI |  |  |
| ＜1300cGy | 1.5 | 0.6-3.8 | 0.389 |  |  |  | 0.8 | 0.3-1.9 | 0.605 |  |  |  |
| ≥1300cGy | 0^a^ |  |  |  |  |  | 0^a^ |  |  |  |  |  |
| GTV (cm^3^) |  |  |  | NI |  |  |  |  |  |  |  |  |
| ＜170.6cm^3^ | 1.1 | 0.4-2.6 | 0.913 |  |  |  | 0.8 | 0.3-1.9 | 0.622 |  |  |  |
| ≥170.6cm^3^ | 0^a^ |  |  |  |  |  | 0^a^ |  |  |  |  |  |
| CD4 M1 ＜268 | 6.7 | 2.3-19.9 | 0.001 | 2.4 | 0.4-12.9 | 0315 | 47.6 | 13.3-170.0 | 0.000 | 24.1 | 2.4-246.1 | 0.007 |
| ≥268 | 0^a^ |  |  | 0^a^ |  |  | 0^a^ |  |  | 0^a^ |  |  |
| CD4 M2 ＜124 | 3.6 | 0.9-14.2 | 0.072 | 2.2 | 0.4-14.0 | 0.398 | 86.1 | 20.1-369.5 | 0.000 | 40.4 | 5.6-289.7 | 0.000 |
| ≥124 | 0^a^ |  |  | 0^a^ |  |  | 0^a^ |  |  | 0^a^ |  |  |

^a^ This parameter is set to zero.

Abbreviations: UVA, univariate analysis; MVA, multivariate analysis; OR, odds ratio; NI, not included in the multivariate regression model; LYM, lymphocytes; MLD, mean lung dose; GTV, gross tumor volume.

Characteristics were entered into the multivariate model in a stepwise fashion if *P*<0.15 and were removed at any point if *P* was >0.15.
