# Supplementary material for: Early variations in lymphocytes and T lymphocyte subsets are associated with radiation pneumonitis in lung cancer patients and experimental mice received thoracic irradiation
Source: Cancer Med. 2020 Mar 24;9(10):3437–44. doi: 10.1002/cam4.2987 (PMC7221303; doi:10.1002/cam4.2987)
Supplement: Supplementary file 2 — Fig S1‐S2 [file CAM4-9-3437-s002.docx]

**Supplementary Figures**

**

**

**Supplementary Figure1.** Mean numbers of lymphocytes (LYM) after RT based on RP classification (GR, grade). Bars indicate 95% CIs. * *P*＜0.001; ** *P*＜0.001.

**

**

**Supplementary Figure2.** Mean numbers of CD4+ based on RP classification (GR, grade). Bars indicate 95% CIs. * *P*＜0.001; ** *P*＜0.001.
